# Case for diagnosis. Verrucous plaque on the pubic region^☆^^☆☆^

**DOI:** 10.1016/j.abd.2020.11.002

**Published:** 2020-11-20

**Authors:** Diego Henrique Morais Silva, Anna Karoline Gouveia de Oliveira, Neusa Yuriko Sakai Valente, Thais do Amaral Carneiro Cunha

**Affiliations:** Department of Dermatology, Hospital do Servidor Público Estadual de São Paulo, São Paulo, SP, Brazil

**Keywords:** Adenoma, Muir-Torre syndrome, Sebaceous glands

## Abstract

Muir-Torre syndrome is a rare, autosomal dominant genodermatosis, characterized by sebaceous neoplasms and visceral carcinomas. The authors describe the case of a patient who, 16 years after the diagnosis of colon carcinoma, presented a verrucous plaque on the pubic region, histopathologically compatible with sebaceous adenoma. The need to investigate this syndrome is emphasized, especially in cases of sebaceous neoplasms located outside the head, face, and neck. Screening for neoplasms in these patients and their families is mandatory.

## Case report

**Figure 1:**
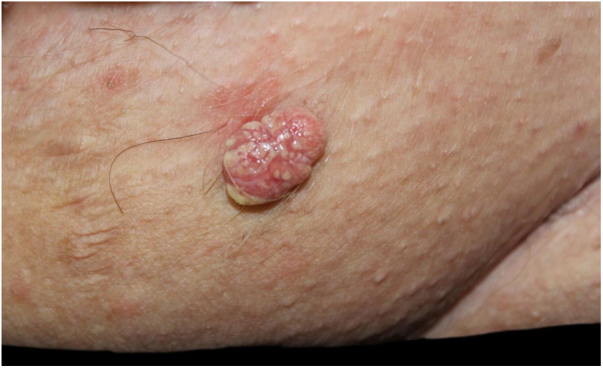
Erythematous verrucous plaque with yellowish structures in the pubic region.

**Figure 2:**
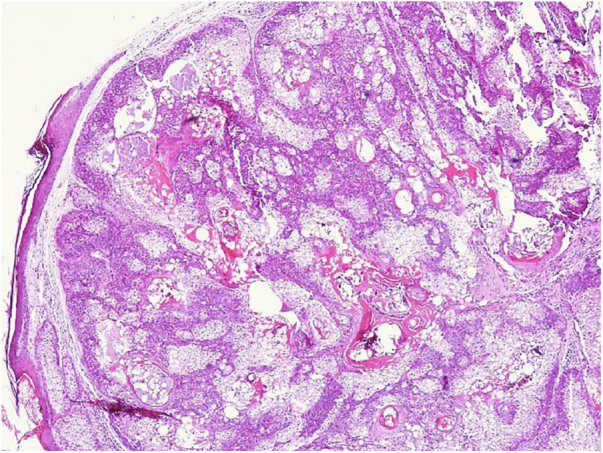
Panoramic view of dermal lobular proliferation composed predominantly of mature sebaceous cells (Hematoxylin & eosin, ×40).

**Figure 3:**
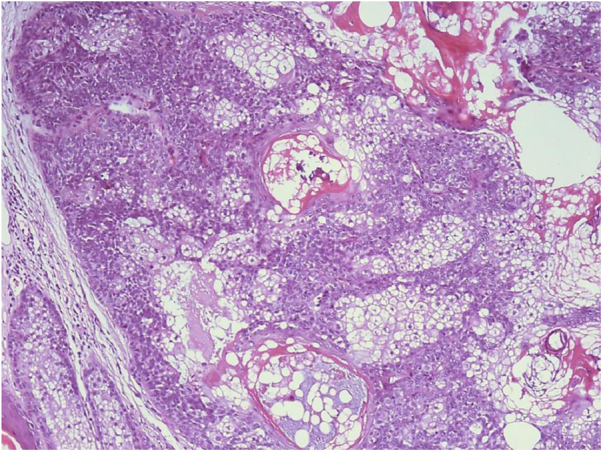
Detail of lobular proliferation with multivacuolated cells in the center and smaller, non vacuolated cells at the periphery (Hematoxylin & eosin, ×200).

The authors describe the case of a 79-year-old woman, presenting a warty, erythematous, and painless plaque for two years on the pubic region (Fig. 1). There were two similar lesions on the face. Dermoscopy showed yellow globules on a milky-red background. She had a personal history of ascending colon cancer 16 years ago, and papillary bladder carcinoma 24 years ago. Her mother died from colon cancer. The lesions were excised, and the histopathological analysis revealed a lobular neoplasia connected to the epidermis with numerous cells with multivacuolated cytoplasm and smaller, non vacuolated cells at the periphery of the lobules (Figure 2, Figure 3).

## What is your diagnosis?

a) Amelanotic melanoma
b) Condyloma acuminatum
c) Squamous cell carcinoma
d) Sebaceous adenoma

## Discussion

Based on clinical and histopathological findings, the authors reached the diagnosis of sebaceous adenoma in a patient with Muir-Torre syndrome (MTS). Sebaceous tumors are rare, most commonly located on the face and neck of the elderly.^1^ There are no specific findings at dermoscopy, although yellowish globules on a milky-red background are common findings in neoplasms with sebaceous differentiation.^2^, ^3^ They are rarely multiple or found below the head, face, and neck; in these cases, the possibility of MTS increases.

The diagnosis of sebaceous neoplasia makes MTS screening imperative. This is a rare variant of Lynch syndrome, autosomal dominant, characterized by the occurrence of sebaceous gland tumors (adenoma, sebaceoma, or sebaceous carcinoma) associated with visceral neoplasia. Among the sebaceous tumors related to MTS, adenoma is the most common, histologically characterized by lobular proliferation with predominance of mature sebocytes in the center and a minority of basaloid germ cells at the periphery. Colon adenocarcinoma is the most common associated visceral neoplasia, especially when proximal to the splenic flexure (unlike sporadic cases), followed by tumors of the genitourinary tract.^4^ The mutations responsible for the syndrome occur in the DNA repair genes: MLH1 (MutL Homolog 1), MSH2 (MutS Homolog 2), MSH6 (MutS Homolog 6), and PSM2 (postmeiotic segregation increased 2).^4^, ^5^ The repair genes encode proteins responsible for identifying and correcting errors during DNA replication. The loss of the expression of the product of these genes can be evidenced through immunohistochemistry, one of the tools used to confirm MTS. The most frequent mutations occur in the MSH2 gene, a finding observed in 90% of MTS patients.

The diagnosis of MTS is clinical, by finding sebaceous neoplasia and at least one visceral tumor without other contributing factors, such as radiotherapy or immunosuppression. Immunohistochemistry and molecular analysis are indicated in cases where there is no family or personal history of visceral neoplasms.

The authors report a case of sebaceous adenoma with unusual location in the pubic region in a patient with colon and bladder cancer. The importance of investigating systemic neoplasms associated with SMT and the monitoring of these patients and their families is emphasized.

## Financial support

None declared.

## Authors’ contributions

Diego Henrique Morais Silva: Approval of the final version of the manuscript; design and planning of the study; drafting and editing of the manuscript; collection, analysis, and interpretation of data; intellectual participation in propaedeutic and/or therapeutic conduct of studied cases; critical review of the literature; critical review of the manuscript.

Anna Karoline Gouveia de Oliveira: Design and planning of the study; collection, analysis, and interpretation of data.

Neusa Yuriko Sakai Valente: Approval of the final version of the manuscript, effective participation in research orientation, intellectual participation in propaedeutic and/or therapeutic conduct of studied cases, critical review of the manuscript.

Thais do Amaral Carneiro Cunha: Approval of the final version of the manuscript, intellectual participation in propaedeutic and/or therapeutic conduct of studied cases.

## Conflicts of interest

None declared.
